# Annual Variation in the Levels of Transcripts of Sex-Specific Genes in the Mantle of the Common Mussel, *Mytilus edulis*

**DOI:** 10.1371/journal.pone.0050861

**Published:** 2012-11-30

**Authors:** Sandhya Anantharaman, John A. Craft

**Affiliations:** Department of Life Sciences, Glasgow Caledonian University, Glasgow, United Kingdom; Argonne National Laboratory, United States of America

## Abstract

*Mytilus* species are used as sentinels for the assessment of environmental health but sex or stage in the reproduction cycle is rarely considered even though both parameters are likely to influence responses to pollution. We have validated the use of a qPCR assay for sex identification and related the levels of transcripts to the reproductive cycle. A temporal study of mantle of *Mytilus edulis* found transcripts of male-specific vitelline coat lysin (VCL) and female-specific vitelline envelope receptor for lysin (VERL) could identify sex over a complete year. The levels of VCL/VERL were proportional to the numbers of sperm/ova and are indicative of the stage of the reproductive cycle. Maximal levels of VCL and VERL were found in February 2009 declining to minima between July – August before increasing and re-attaining a peak in February 2010. Water temperature may influence these transitions since they coincide with minimal water temperature in February and maximal temperature in August. An identical pattern of variation was found for a cryptic female-specific transcript (H5) but a very different pattern was observed for oestrogen receptor 2 (ER2). ER2 varied in a sex-specific way with male > female for most of the cycle, with a female maxima in July and a male maxima in December. Using artificially spawned animals, the transcripts for VCL, VERL and H5 were shown to be present in gametes and thus their disappearance from mantle is indicative of spawning. VCL and VERL are present at equivalent levels in February and July–August but during gametogenesis (August to January) and spawning (March to June) VCL is present at lower relative amounts than VERL. This may indicate sex-specific control mechanisms for these processes and highlight a potential pressure point leading to reduced reproductive output if environmental factors cause asynchrony to gamete maturation or release.

## Introduction

Common mussels (*Mytilus edulis, Mytilus galloprovincialis and Mytilus trossulus*) are sessile, intertidal, filter feeding, sedentary organisms that inhabit coastal areas over a wide geographical area. Their habitat and ecology result in the accumulation of contaminants within their tissues (pesticides, hydrocarbons, metals etc.) [1], [2], [3], [4], [5], [6] and this can result in a “stress syndrome”. Chemicals may be determined directly or their biological effects can be measured by a number of indices (whole animal, cellular, molecular) [7], [8], [9], [10], [11]. The common mussel is thus a useful sentinel as a biological indicator of marine pollution and has been widely used in bio monitoring programmes like ‘mussel-watch’ [1], [12], [13], [14], [15], [16] and other research programs [1], [4], [8], [17], [18].

In previous studies the gender of the mussels and the stage of the reproductive cycle, were not necessarily considered to be variables that would affect the outcome of the investigations. However, many studies have identified sex-specific differences in the metabolism of xenobiotics in mammals [19], [20] but also in mussels [21], [22]. In part, ignoring gender in mussel-studies was a consequence of the difficulty of sex identification. Mussel is gonochoristic but has no external sign of dimorphism, either morphological or anatomical. Historically, the only reliable method for gender determination was time-demanding histology but this is only informative during certain stages of the reproductive cycle when gametes are visible in the gonad (mantle). For that reason we developed a RT-PCR-based assay for sex-identification and showed that the method was more accurate than alternative approaches [23]. Subsequently we have improved the approach by the introduction of a Quantitative, Real-time PCR assay and shown that the method is accurate and applicable through all of the times tested during the reproductive cycle [24].

The method for sex determination is based on the presence of sex-specific transcripts in mantle. The targets for this are vitelline coat lysin [VCL] [25] and vitelline envelop receptor for lysin [VERL] [26] which play key roles in fertilisation allowing sperm to enter the egg by the sperm-located VCL binding to egg-located VERL. Not only do these transcripts allow sex identification but they can also be used to follow the events of reproduction. The reproductive cycle in mussel has four distinct phases in which: (a) gametes develop; (b) gonadal follicles are filled with ripe gametes; (c) spawning; and (d) the resting or ‘spent’ stage [27]. In our earlier study [24] both VERL and VCL transcripts were present in the mantle at peak levels in March (assumed to be stage b) and declined thereafter to minimal levels in June to August (assumed to be stage d) at which point they started to increase again. However, the study was incomplete in two respects. Firstly the stages of the reproductive cycle were not confirmed through e.g. histology and thus the transcript levels were not anchored on physiological events and the analysis was incomplete with samples taken in only 11 months of the 21 month study.

The signals for the initiation of gametogenesis, the control of gamete production and spawning in mussel are unknown. Environmental factors such as temperature may be cues for biological control processes and by comparison to higher organisms those controls may involve steroid signalling. The role of steroid hormones in molluscs has been controversial but definitive evidence has been presented for the occurrence of oestrogens, androgens and progestins [28], [29] and steroid biosynthetic enzymes [30], [31] in various species including mussel. Serotonin is involved in various events of gamete development in molluscs and evidence has been presented for cross-talk between this regulator and oestrogens in the Japanese scallop, *Patinopecten yessoensis* [32]. Potentially, steroid receptors may be involved as regulators of gametogenesis and for that reason we have considered the oestrogen receptors. Two oestrogen receptors (ER1 and ER2) are found in molluscs but they are unlike those of higher organisms since the distinctive ligand-binding domains (LBDs) activate transcription constitutively, without added ligand [33]. The variation of ER2 transcripts over the reproductive cycle of mussel has been the object of a previous study and was found to have peaks at April and October with lows at January and July [34]. However, that study was of mixed-sex animals and it is possible that the expression follows distinctive sex-specific patterns and that the role of ERs is different in each sex as found in mammals [35], [36].

The study reported here had the aims of determining levels of relative expression of VCL and VERL by sampling at monthly intervals over 13 months and thus confirming the validity of the method for sex determination over a continuous annual cycle. Histology of each mussel sample was also conducted so as to anchor the expression data with stages of the reproductive cycle. Water temperature was also considered as a potential environmental regulator. Further, to start to unravel the regulatory aspects of the cycle the relative levels of two other transcripts were determined. These were ER2 and a cryptic transcript termed H5. H5 is a female-specific transcript, of unknown function obtained from the same SSH library that provided VERL (Kennedy, Dempsey and Craft, unpublished). Additionally, another aim was to determine the cellular locations of the 4 target transcripts in the gonad using induced spawning of ripe animals to collect sperm and eggs.

## Materials and Methods

### Ethics Statement

No specific permissions were required for the collection by hand of naturally occurring shellfish from the shoreline at the Lunderston Bay location. The site is public and part of the Muirshiel Regional Park and not a protected area. The only species sampled was *Mytilus edulis*, the common mussel and did not involve any protected or endangered species.

### Mussel Collection, Dissection and Artificial Spawning

Mussels (*Mytilus edulis*) (>3 cm in length) were collected at the same calendar date at monthly intervals at low tide from inter-tidal rocks at Lunderston Bay on the Firth of Clyde, UK (55°55′53 North, 04°53′07 West) between February 2009 to February 2010. The animals were transferred to the laboratory in sea water and portions of mantle were dissected and used for RNA isolation and histology. Sex for each animal was confirmed by histology, by the presence of ovarian follicles or sperm tubule lumen in the mantle matrix using the method previously described [24]. Species was confirmed by generation of the 128 bp Glu PCR product (data not shown) [37].

The animals for the artificial spawning experiment were collected from the same location in March 2011, transported to the laboratory and placed in tanks of sea water at 8°C until they were induced to spawn. The heat shock method explained in the past [38] was used for the artificial spawning with an additional mechanical stress application, for quicker initiation of spawning, suggested by Michael Moore (Plymouth Marine Laboratory, UK). For artificial spawning, mussels were scrubbed to remove all the byssus thread and symbiotic organisms. Each mussel was then placed separately into a 250 ml beaker with 150 ml of filtered (12 µm filter) and UV-sterilised sea water. These beakers were then placed on a shaking platform at 8°C for 30 minutes. The mussels were then placed in a closed plastic bucket individually and given mechanical stress by shaking them gently for several minutes before transferring into a beaker with 150 ml of filtered and UV sterilised sea water at 28°C. In most cases, mussels spawned within 20 to 40 minutes after the mechanical and heat shock. Once initiated spawning was allowed to continue for about an hour and then the animals were dissected and sections of mantle were collected for RNA extraction and histology. A drop of sea water containing gametes from the spawned mussel was examined under the microscope. The gametes were collected from the remaining water in a 50 ml Falcon tube centrifuged at 2°C for 15 minutes at 1000 g. The pellets with the gametes were washed with 1 ml of UV- sterilised, filtered sea water and then transferred into a 1.5 ml micro centrifuge tube. The gametes were pelleted and a portion of the pellet used for extraction of RNA. The rest was concentrated with a cytospin device (5 mins×1000 g) onto a microscope slide prior to fixation and staining for microscopy.

### Water Temperature

Water temperature data (2000–2010) was collected by the University Marine Biological Station Millport (UMBSM), Firth of Clyde in two ways. For 2000–2010 monthly means were derived from daily mercury-in-glass thermometer readings of seawater drawn in a bucket from the sea surface at 0900 hours on a daily basis (Monday to Friday). For data for Feb-09 to Feb-10 temperatures were recorded by half-hourly readings (data not shown) using Vemco temperature loggers on behalf of Marine Scotland, Aberdeen Marine Laboratory and daily means were calculated. The daily figures have been used to generate monthly means for the period of interest. All measurements were made at Keppel Pier at the UMBSM (55°55′30 North 05°54′00 West) which is 19.5 km from Lunderston Bay.

### RNA Isolation

RNA was extracted from ≈50 mg of mantle tissue using Trizol (Invitrogen, UK #15596-18) following the standard manufacturers’ protocol with an rDNase step (Ambion, UK #AM1906). The RNA was quantified and checked for quality using Nanodrop (ND-1000 Spectrophotometer) and stored at −80°C. For the extraction of RNA from the gametes the Trizol was pre-heated to 65°C, 1 ml was added to each preparation and incubated at room temperature for 30–45 minutes prior to the standard protocol. Since the RNA from the gamete samples had very low concentration, they were then concentrated using the RNA Clean-up kit (Zymo-Research, US #R1015) with the standard manufacturers’ protocol.

### cDNA Synthesis and PCR

qPCR assays have been performed using the same amount of cDNA for all four genes, the male specific VCL (Accession# FM995162), the female specific VERL (Accession# FM995161), H5 (Accession# JX297444) and the estrogen receptor ER2 (Accession# AB257133) using Actin (Accession# AF157491) as an endogenous reference gene.

cDNA was produced from RNA extracted from mantle or gametes using SuperscriptIII reverse transcriptase (Invitrogen, UK #18080-044) by the manufacturer’s standard protocol. The cDNA was then diluted 1∶10 using RNase free water and used as a template for qPCR. qPCR was performed in duplicate for all target genes as previously [24] with samples collected for the temporal study (24 animals per month) and from the artificially spawned mantle tissues and gametes.

The Platinum SYBR green qPCR super mix (Invitrogen, #11733-046) was used with the following primers:

VCL-F 5′AGAGCTGTTTTGGCCACAGT3′,

VCL-R 5′ TTGCGTTTGACATGGTTGAT3′;

VERL-F 5′CCGAAGGAAATGGAACTGAAA3′,

VERL-R 5′CCCTGCAATCGTATGGAATC3′;

ACT-F 5′AGGACTTGTAACCACC3′,

ACT-R 5′CACCGATCCAGAGTAT 3′;

H5-F 5′AAGGATATCGACGCATACGCTGCT 3′,

H5-R 5′TTTCGTCTCGGCTTGACCCTTCAT 3′.

The primers used for determination of ER2 were as described [34]:

ER2_FP (5′ GGA ACA CAA AGA AAA GAA AGG AAG 3′) and

ER2_RP (5′ GCT GGA TTA GGA CTG CCA CTT G 3′).

Efficiency of qPCR reactions were: VERL 95.3%; VCL 97.3%; H5 91.8%; actin 97.9%; ER2 92.9%.

### Data Analysis and Statistics

Ct values were determined by setting a constant baseline, for all samples and the ΔCt was calculated with respect to actin. Sex was determined calculating the intra animal ΔCt (VCL – VERL) and a negative value indicated male and positive indicated female. Relative expression levels were determined using an arithmetic comparative 2^−ΔΔCt^ method [39]. The ΔCt were initially calculated as [Ct (Gene-Of-Interest) – Ct (Actin)] where Gene-Of-Interest was VERL, VCL, H5 or ER2. Then ΔΔCt was calculated [(Mean ΔCt) s_ample_ – (Mean ΔCt) _Calibrator_]. The mean ΔCt for VERL from females collected in February 2009 was used as the calibrator for VERL, VCL and H5. Thus ΔΔCt (Gene-Of-Interest)_ any month_ = ΔCt (Gene-Of-Interest)_ any month_ – mean ΔCt (VERL)_ Feb09_ so for VERL in Feb 09 ΔΔCt = 0 and 2^−ΔΔCt^ = 1. Sex-specific analysis was performed to determine the relative transcripts of ER2 in both male and female samples. ΔΔCt was calculated, with actin as the endogenous control and with ΔCt (ER2) in female animals February 2009 as calibrator. For the artificial spawning experiment the relative expression level of transcript was calculated using the mean ΔCt (VERL) in mantle of artificially spawned female animals as the calibrator. For ER2 in gametes, the female mantle ER2 value was used as the calibrator for ΔΔCt calculations.

Statistical analysis was carried out to test for significance of difference of all of the target genes, in all preparations, relative to VERL in the peak month (February 2009). To avoid making assumptions on homogeneity of variances and normal distributions the Mann-Whitney U test (p<0.05) was employed using GraphPad Prism software (v5). A standard error for each relative gene expression value [39] was calculated to derive the data variation which was mainly due to biological variation in animals. Within-month comparisons of VCL and VERL were also conducted by Mann-Whitney U test.

### Histology

Sex was determined by the presence of ova or sperm in the mantle of mussels. The mantle tissue was fixed in Davidson’s sea-water fixative which consisted of 3% formaldehyde in filtered, UV-sterilized sea water. The fixed mantle tissue was passed through washes with different concentrations of ethanol (80%, 90% & 100%) and then was molded into paraffin wax blocks and the tissues were sectioned using a microtome and mounted on APES coated slides and stained using the standard H&E staining protocol prior to microscopical examination.

For fixing the gametes the pool of eggs and sperm were collected separately in a centrifuge that was adapted as a cyto-spin. The gametes were loaded on a cyto-spin tube with a pin hole at the bottom and were centrifuged on top of a slide to concentrate all the gametes onto a spot. This spot of cells was then fixed and stained. 1 ml of Davidson sea-water fixative (4% formaldehyde with sea water) was added on the spot of cells. The slide was air-dried in a fume hood for 1 hour, and was washed successively with 80%, 90% and 100% alcohol. After the final wash, the slide was stained with H & E using the normal staining procedure prior to examination under a microscope (100 × magnification).

## Results

### Variation of Transcripts of Sex-specific Genes Over a Reproductive Cycle

**Figure 1:**
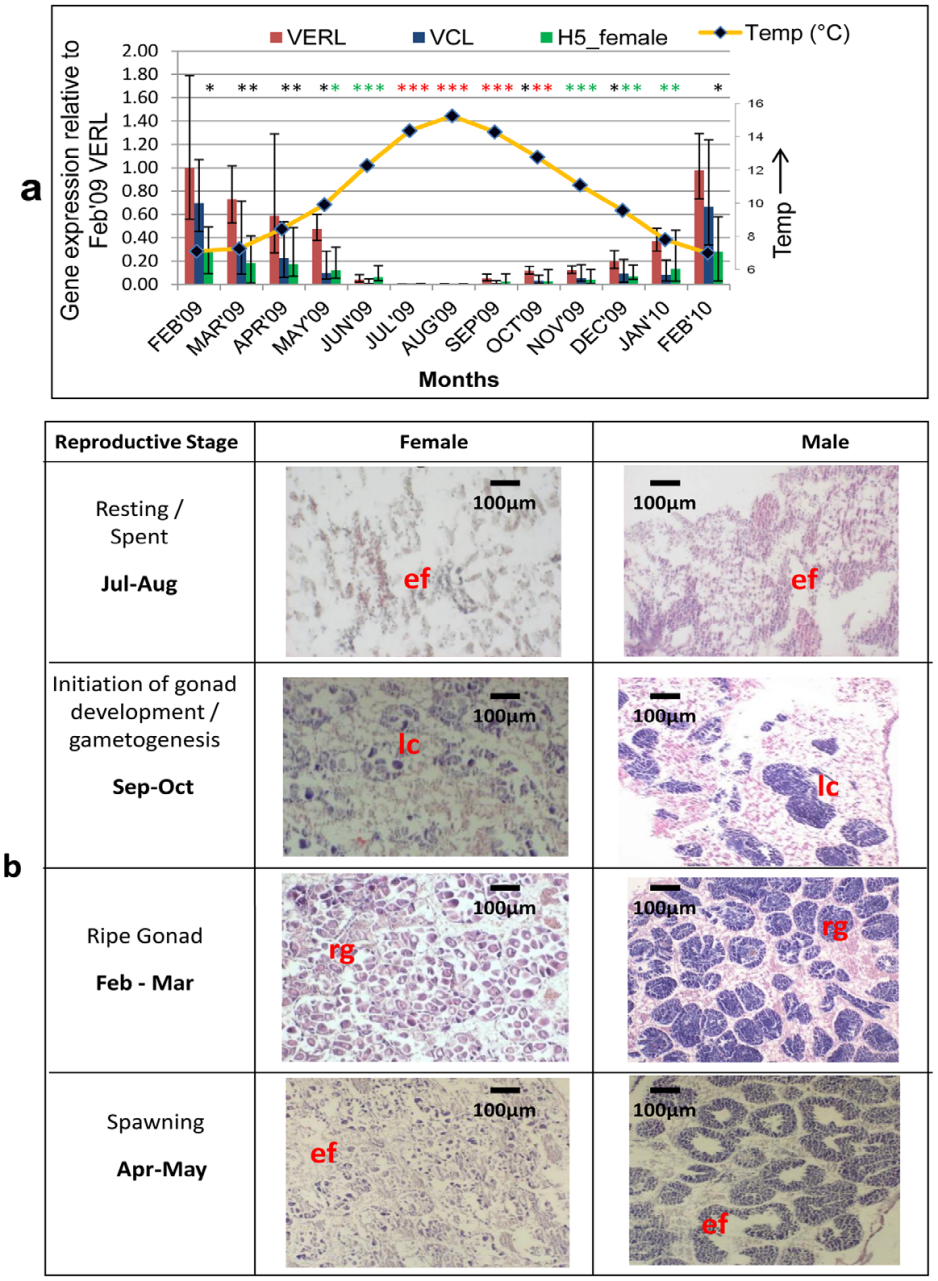
Temporal variation of sex-specific transcripts in mantle in relation to gonad histology in mussel. (a) Relative levels of VCL, VERL and H5 transcripts were determined by qPCR in samples collected at monthly intervals. The results for each target, transcript at each sampling time are computed relative to VERL in female at Feb -09 (see Methods). Data shows mean ± SEM for duplicate determinations with n between 2–17 (male) or 7–20 (female) (See Table S1 for monthly details). Significance; *p<0.05, *p<0.005 and *p<0.0005. Monthly, average water temperatures are also shown. (b) Representative images of the H&E-stained mantle at 20X magnification, from various stages of gametogenesis in mussels during the study period. *Abbreviations* ef - empty follicle or tubule; lc - Leydig cells; rg - ripe gametes.

The relative levels of transcripts for male-specific VCL, female-specific VERL and H5 in mantle were determined relative to the February 2009 female VERL at monthly intervals over a period of 13 months encompassing a complete reproductive cycle (Figure 1a) (number of male and female animals found in each month are shown in Table S1a). Surprisingly the sex ratio was found to be 60∶40, female: male over the entire sampling period. Partial results for VCL and VERL have been previously described [24] and H5 is a cryptic, newly-isolated sequence from a female-specific subtraction library and extended by 3′-RACE to 1100 bp (GenBank JX297444). The identity of H5 cannot be established and BLAST searches barely reach statistical significance but show similarities to a hypothetical gene in Oreochromis niloticus (E = 3e^−7^) and Rapunzel in Danio rerio (E = 4e^−7^) (Table S2a). The relative expression of H5 in male was maximally 3% of matching female samples (May 2010) but more generally less than 0.1% (Table S2b) In the determinations of relative transcript levels, actin was used as the reference gene as it has been observed to be invariant in previous studies [24] and the same was found in the current study. The levels of this transcript did not have any significant variation (Mann-Whitney, p>0.05) in both male and female samples, based on cDNA input, over the reproductive cycle (Figure S1). It is also seen that the actin levels was invariant for the artificial spawning as well (Table S3). Signals for VCL and VERL, with Ct<No template control were found in all within sample comparisons but the sex-specific level was always much higher than the cross-sex-specific signal (e.g. for male ΔCtVCL<<ΔCtVERL) (Table S4). In most female samples, relative levels of VERL>>VCL by three orders of magnitude throughout the sampling period other than the quiescent months of July and August when it is only one order greater. For males relative levels of VCL>>VERL by at least two orders of magnitude, throughout the sampling period, other than July and August when it is only 8 times higher.

The study started in February 2009 which corresponds to the maxima of relative expression of VCL, VERL and H5 (Figure 1a). Subsequently, the levels of all three targets decline reaching minima by July and they remain at the same low levels to August. Subsequently, relative levels of the transcripts start to increase again re-attaining, by February 2010, the same levels seen at the start of the study. In parallel, histology was carried out (Figure 1b) and whenever it was possible to establish sex by this method the result was in total agreement with the molecular result (numbers for male and female animals determined by histology in each month are shown in Table S1b). Histology also allowed the various stages of gametogenesis and the reproductive cycle to be distinguished by the number of developing/ripe gametes seen in the gonad and in Figure 1b those stages vary from follicles being filled with developing/ripe ova and tubules filled with spermatozoa to totally empty gonads with no gametes. Based on visual, qualitative inspection of each individual histology slide, the presence and abundance of gametes was found to be associated to the levels of the transcripts of the sex specific gene in that sample. As VCL and VERL/H5 decrease from February gradually till July gonads have increasing free space month by month and by June gametes are rare in both male and female. Between July and August the mussels are in their resting stage with respect to gametogenesis and presumed to be replenishing energy reserves [27] prior to the initiation of gametogenesis from September. As VCL and VERL/H5 increase from September to February the gametes are seen to be developing month by month. The follicles start filling with ova and are rounded off once filled with ripe gametes. Similarly, the tubules have developing spermatozoa and have developed boundaries filled with mature sperms. From the individual histology slides and qPCR results it is seen that there is an overlap between the stages at any one sampling over the 13 months sampled.

To investigate the effect of environmental conditions on the reproductive cycle and variation in transcript levels, surface water temperature measurements are reported. These were taken at the nearby University Marine Biological Station Millport (UMBSM), Firth of Clyde and are provided as monthly means. During the period for which mussel data was collected, the maximum, monthly, mean water temperature was recorded during August while minima occurred during February 2009 and March 2010 (Figure 1a). Looking at data for previous years, from 2000 (NB this is based on one reading per day rather than 48), maxima in August occurred in 9/11 years (14.7±0.77C, mean ± SD) and in the other 2/11 was in July (14.1±0.89C). Minima were more variable but occurred in February in 6/11 years (7.6±0.9C) while it was in March for 5/11 (7.6±0.9C).

At the February maxima, the relative levels for VCL and VERL transcripts are equivalent while H5 is present at about 30% of VERL. VCL and VERL are also equivalent during the months of minima, June and July. However, at other times of the cycle (spawning, March to May and gonad development, August to January), the intra month comparison shows VCL to be consistently and significantly, lower than VERL (Mann-Whitney U test p< at least 0.05). This is reflected also in the histology since the loss of gametes is more extensive in males at earlier times than females.

### Variation of Transcripts for ER2 Over a Reproductive Cycle and their Sex-specific Associations

**Figure 2:**
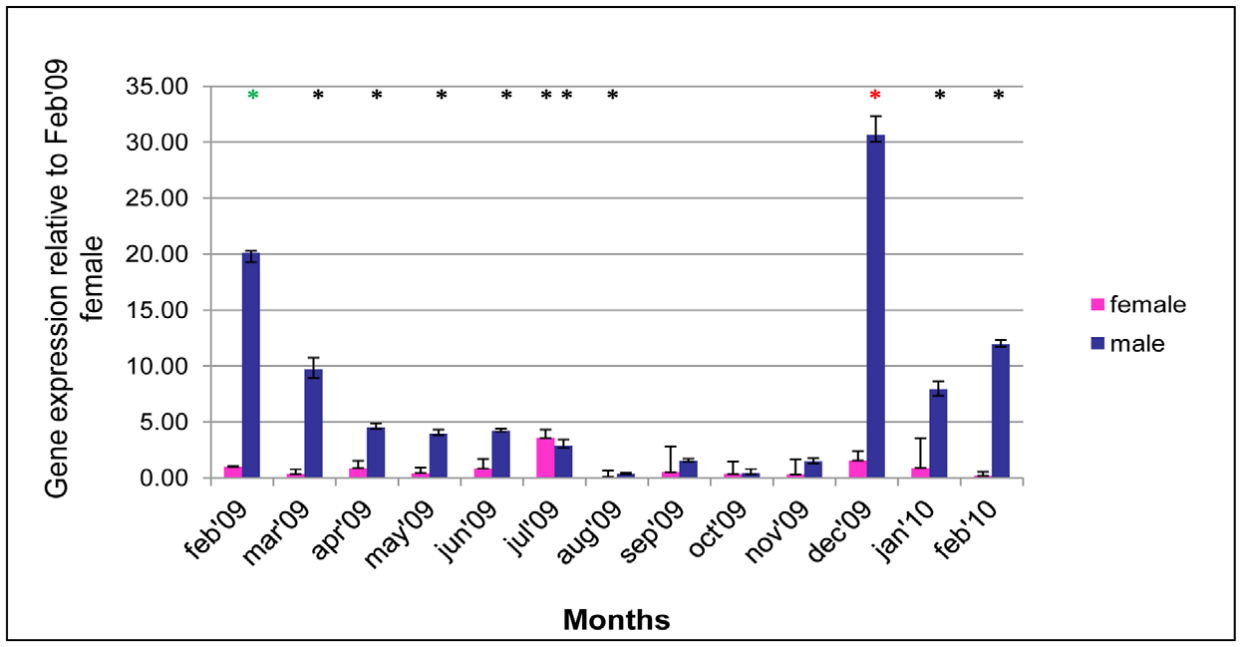
Sex-specific variation of ER2 transcripts in mantle of mussel. Levels of ER2 transcripts were determined by qPCR in samples collected at monthly intervals. Results are computed relative to the mean for ER2 transcripts in female at Feb-09 using the same approach as in Figure 1a. Significance; *p<0.05, *p<0.005 and *p<0.0005.

The variation of the ER2 transcripts in the same samples, over the same period of time is shown in Figure 2. The relative level of the ER2 transcripts in both male and female was always much lover (≤10^−3^) than VERL in Feb’09. For that reason the values reported are based on comparison to ER2 in female animals during February 2009. When the sex-specific analysis was done, it is clear that the level of expression of ER2 is significantly higher in male when compared to female during most months. The statistical test for female animals showed a significantly higher value in July, significantly lower in August but was constant in other months. In males the expression pattern was similar to that of VCL but increasing from a low in September to a peak in December rather than February and there-after levels decrease until August. The intra month comparison shows the expression of ER2 in males to be significantly different to that in females almost throughout the year except for the months from August to November.

### Localisation of the Transcripts within the Mantle and Gametes Using Artificial Spawning

So as to identify the locations of the target transcripts within the mantle, artificial spawning was employed to collect the gametes and check for the presence of VCL/VERL/H5 mRNAs. Sixteen mussels were induced to spawn, of which 7 produced sperm and 5 eggs. Emission of sperm was clearly visible as a white fluid and motile sperm could be observed under the microscope. Emission continued for at least 4 hours but the samples for analysis were collected after 1 hour of the start of emission of gametes. Thus analysis of mantle is after partial spawning and this can be seen in both the histology and qPCR results of the spawned mussel mantle. The other 4 animals did not spawn at all. When the non-spawning mussels were dissected and the mantle histology was conducted, there were very few ripe gametes in their gonads. One of them was a male that had tubules with very few sperm and the other 3 were females with largely empty follicles and very few ripe eggs (data not shown).

**Figure 3:**
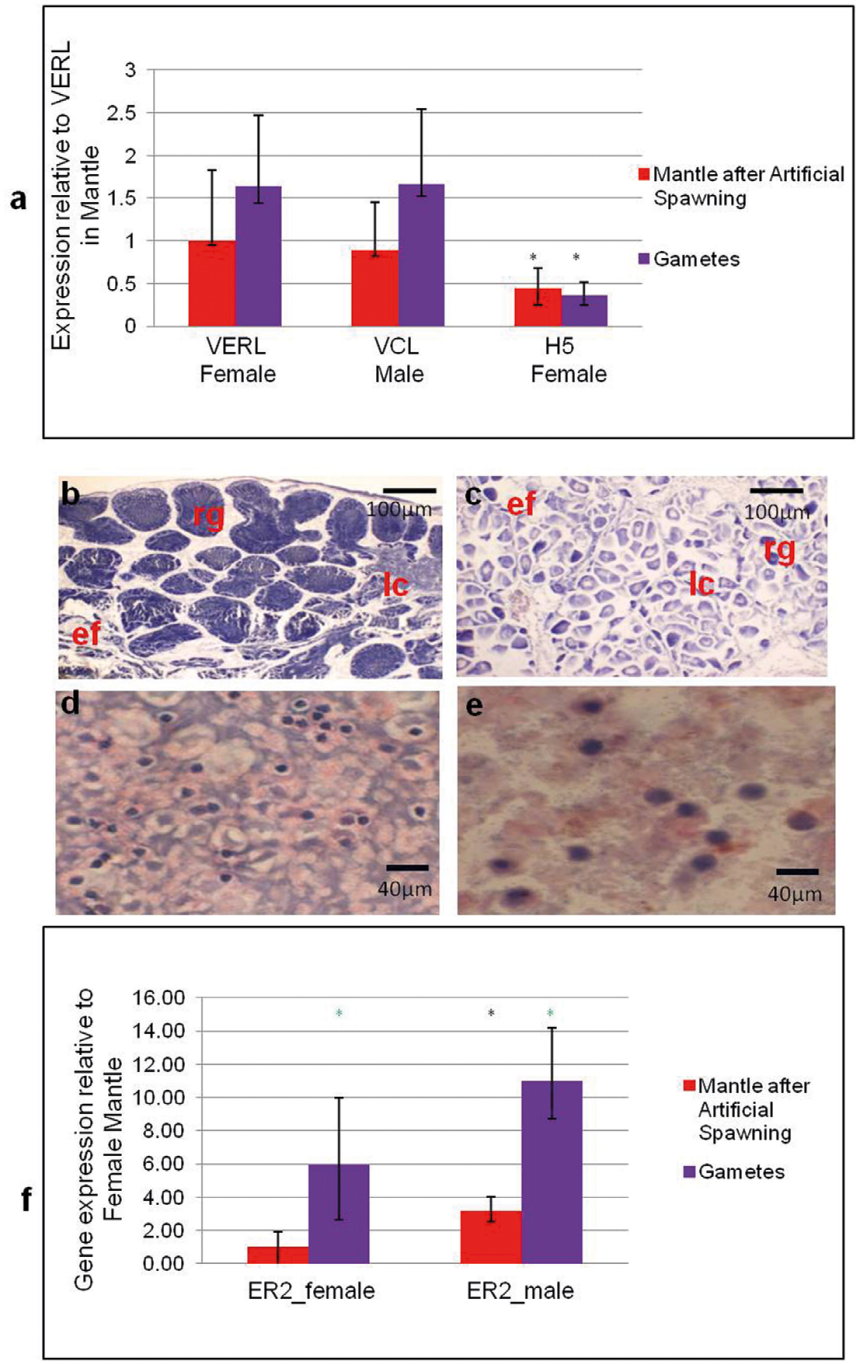
Analysis of gametes and mantle for sex-specific transcripts after artificial spawning. (a) Levels of VCL, VERL and H5 were determined by qPCR in gametes and mantle after artificial spawning. Levels of each target are computed relative to VERL in mantle of the artificially spawned, female animals. (b) and (c) H&E stained sections of male and female mantle (respectively) at 40X magnification after artificial spawning. Abbreviations, ef - empty follicle or tubule; lc - Leydig cells; rg - ripe gametes. (d) and (e) H&E stained gametes, sperm and ova respectively at 100X magnification collected from the artificially spawned mussels using a cyto-spin. Tails of the sperm and in ova a pink cytoplasmic layer and a purple nucleus can be seen. (f) Levels of ER2 transcripts were determined as in (a) but are relative to ER2 in female mantle after artificial spawning. Significance (Mann Whitney); *p<0.05 and *p<0.005.

The relative levels of VCL, VERL and H5 were determined in the artificially spawned gametes. Results are shown in Figure 3a and expressed relative to levels in the artificially spawned female mantle. Each of the transcripts was present in gametes and matching mantle in a sex-specific manner (VCL was not present in eggs and VERL and H5 was absent from sperm). The levels of VCL and VERL/H5 appeared to be enriched in the gametes but this did not reach statistical significance.

Histological examination of the mantle of post-spawning animals showed that the mussels had not released all of their gametes (Fig. 3b & 3c) and many ripe gametes remained in the gonads. However, when compared to mussels collected at the same time that had not been induced to spawn (data not shown), there was a difference in anatomy with the appearance of dissolved follicle and tubule boundaries and the presence of empty follicles and tubules in female and male mussels subjected to artificial spawning respectively. The gametes were collected and examined by histology and sperm and eggs were easily identified and differentiated. The H&E stained samples show that the sperms are oval in shape and have a tail while the eggs are larger and contain a round nucleus (purple) and surrounded by cytoplasm (pink) [Figure 3d & 3e]. The number of eggs collected was relatively low when compared to the number of sperm.

The relative levels of ER2 transcripts in the same preparations were also determined and are shown in Figure 3f. The result is calculated with respect to ER2 transcript in matched female mantle. It is seen that the gametes and mantle contain these transcripts and also there is a sex specific difference in the level of expression of ER2 and the expression is significantly higher in sperm when compared to eggs. The enrichment of ER2 transcripts in the gametes is also significant and higher than ER2 expressed in artificially spawned mantle, which is in contrast the situation to VCL, VERL/H5 for this analysis. The level of ER2 in spawned male mantle is seen to be significantly higher than female mantle.

## Discussion

The common mussel has been used world-wide as a sentinel of pollution, of environmental health and by association human health [1], [4], [8], [12], [13], [14], [15], [16], [17]. In the majority of these activities, sex of the mussels has not been determined and thus variation is likely to occur from study to study because of differences in the gender balance. Early work has indicated the significance of gender in the metabolism of xenobiotics in digestive gland of mussel [21], [22]. Similarly the possible interaction of the reproductive stage has often been disregarded. A recent study has investigated the effect of gender on transcript abundance in digestive gland of *M. galloprovincialis* and discovered significant differences. Genes concerned with carbohydrate metabolic processes, translation, catabolic processes and others were expressed at differential, sex-specific levels and these were also influenced by seasonal factors[40]. However, it was concluded that the significant factor with respect to season was chitin metabolic processes rather than gonadal development. The importance of considering the stage of gonadal development is highlighted by a study also of *M. galloprovincialis* that demonstrated a seasonal variation in organochlorine contaminants in whole mussel [5]. Further such variation was associated with gonad developmental index and lipid content.

We have highlighted the importance of establishing sex of mussel and have developed a method for sex determination based on molecular methods detecting sex-specific genes. The study reported here has confirmed and extended our earlier work [23], [24] by showing that the abundance of the same genes can be used to follow all of the reproductive stages. It demonstrates that over a continuous, year-long period the transcripts of VERL and VCL can be used to identify sex in mussel even at stages of the reproductive cycle when the gonad is ‘spent’ or developing. Surprisingly the number of females in the samples exceeded males to a significant extent (60∶40). Such variation in the sex ratio, away from 50∶50 was not observed in the previous study with the same sample number from the same site [24]. It is not clear at present whether this could be an accident of sampling or a real shift in the population at the site. Actin is used as the endogenous control and the level of this transcript is seen to be constant over the year, in agreement with the earlier work but in contrast to the situation found in another study [41]. In that study actin did vary with developmental stage and there is no obvious answer to the discrepancy. In addition to the qPCR results, evidence is now provided through histology that the relative levels of these transcripts are directly related to the presence and number of eggs and sperm in the gonads.

We have demonstrated that the transcripts for VERL, H5 and VCL are present in the gametes. The data does not exclude the possibility that these mRNAs are present in the gonad in both gametes and somatic cells but the correlation between transcript level and gamete numbers through this period suggest that they are indeed restricted to the gametes. The function of the protein products of these genes is in the mature sperm and egg [42] so exporting the proteins from somatic cells to gametes would call for a complex and unnecessary process. Assuming that the hypothesis that the transcripts are present solely in the gametes is correct, their relative levels in mussel gonad between February and July is a proxy for the extent to which spawning has occurred. Within the resolution of the sampling frequency, a single cycle of gametogenesis occurs starting in August/September and reaching its conclusion in February. This is in contrast to previous views that, in the mussel, there are multiple cycles of gametogenesis [18], [27]. Spawning is successive and appears to be by repeated, partial events that occur starting in February and completed by July. This duration of spawning is considerably longer than that recorded for *M. edulis* at other geographical locations [43].

Mussels reproduce by broadcast-spawning into the sea and males and females are presumed to be synchronised to produce mature gametes at the same time point and to spawn simultaneously but environmental cues for the two processes and the mechanisms of control are not well characterised. In other molluscs environmental factors including water temperature, day length/ambient temperature, food availability, stage of the tidal and lunar cycle and physical agitation [43], [44], [45], [46], [47] play part(s) in these processes. In the study reported here, there is an apparent association between maximal water temperature and initiation of gametogenesis and minimal water temperature and initiation of spawning. The gradient of water temperature may thus be important in transitions of the reproductive stage but we cannot exclude the role of photo-period since the transitions of water temperature occur as day-length shortens in autumn or lengthens in spring. The relative role of water temperature and photo-period can be tested by future experimentation.

A number of neuropeptides and a neurotransmitter have been implicated in gamete development and spawning in other molluscs [32], [47], [48], [49], [50], [51] but the involvement of homologues of these molecules in reproductive biology of mussel is unknown. A number of studies in mussel have highlighted the possible role of serotonin and its receptor 5HTR which may be regulated by oestrogens. Serotonin is involved in the spawning process, stimulating terminal maturation and release of oocytes in scallop (*Patinopectin yessoensis*) [32] and zebra mussel (*Dreissena polymorpha*) [50] and spermatozoa in zebra mussel [52]. Transcripts for a 5HT1-like receptor (5HT_py_) have been located in oocytes and gonoduct in females but are restricted to gonoduct and a sub-group of spermatids in male scallop (*Patinopectin yessoensis*). In this respect it is interesting that oestrogen up-regulated 5HT_py_ transcript in ovary but not testis [32]. Dimorphic control by serotonin in the two sexes is also indicated by the observation that metergoline (a 5HTR antagonist), at low concentration, stimulates spawning in male zebra mussel without effect on female [50].

Various observations suggest that spawning in mollusks is influenced by prostaglandins possibly by modulating the effects of serotonin. In the scallop, *P yessoensis*, serotonin-induced oocyte release was enhanced by the presence of PGE_2_ but the prostaglandin had no effect alone [53]. An association between levels of PGE_2_ and PGF_2a_ and spawning were observed in the hermaphrodite scallop, *Argopecten purpuratus* with increasing levels during spawning but diminished once spawning was completed [54]. Recent observations suggest that PGF_2a_ has a distinct role that opposes the effects of PGE_2_ because it blocked the movement of eggs through the gonoduct of *P. yessoensis* [55]. The two prostaglandins may thus play an important role in regulating sequential spawning events. Intriguingly oestrogens may regulate prostaglandin synthesis in *P. yessoenensis* [56] providing a further putative link between these steroids, serotonin and prostaglandins in the regulation of spawning in mollusks. With respect to *Mytilus species*, indirect evidence suggests that prostaglandins are involved in spawning in *Mytilus californianus* since UV- and H_2_O_2_-induced spawning was inhibited by the COX inhibitor, aspirin [57]. The aspirin-sensitive synthesis of prostaglandin-like compounds in *Mytilus edulis* has been described but definitive identification of the compounds was not provided [58] and no direct studies of the role of PGs on spawning in this species has been conducted.

The possible role for steroids and particularly oestradiol in these processes has been much discussed [59]. Even though molluscan ERs do not appear to bind oestrogens [33], their potential role in gametogenesis in mussel is interesting and we have investigated the temporal variation in the level of ER2 transcripts. To the best of our knowledge this is the first time that expression of transcripts for ER2 have been investigated in separate sexes of mussel and their presence differs between the sexes in quantity and temporal pattern. A previous study [34] of the natural variation of ER2 noted temporal variation of expression of this gene with stage of gametogenesis but used mixed sex animals and thus it is not possible to compare the two sets of data. In *M. edulis*, ER2 is expressed at higher levels in male compared to female suggesting a role such as is seen in higher organisms for testicular development [35]. The role of ER2 in testicular maturation is also indicated by the observation that peak level of this transcript is in December, before peak level of VCL and prior to the start of spawning. However, ER2 is present in artificially spawned spermatozoa implying a potential role in post-spawning events as well as in spermatogenesis. An apparently different result was reported in another study with ER greater in ‘developing’ (collected February) compared to ‘mature’ (collected April) testis [59]. However, ‘developmental stages’ were not fully defined and if mussels in the south of England follow a similar reproductive cycle as those in the Clyde the mussels described as mature are likely to be post spawning and the ER to have decreased because of sperm located transcript. In contrast to the male, in female, ER2 only rises above, what is otherwise, a relatively constant level during July and this coincides with presumed initiation of oogenesis. Higher levels are also seen in December and January but these do not reach statistical significance. Thus it may be that ER2 is primarily involved in oogenesis at the outset of the annual process and plays a minimal role thereafter. These observations do not provide a link between possible interactions between oestrogens, oestrogen receptors and serotonin in the control of spawning. They do however, strongly indicate differences in mechanisms between male and female mussel in the cross-talk of these putative regulators.

It is noteworthy that the transcripts for VERL and VCL, present in gametes, attain equivalent peak values (February) and reach minimum expression levels (July) at the same time points. However, the kinetics of their appearance and subsequent decline are different in a sex-specific manner. It is seen that VERL in the developing ovary and spawning animal has a wider window in female when compared to VCL in male. These observations at the transcript level are matched by histology. The difference in rate of gamete development and of spawning noticed between the male and female could have consequences for reproductive success. In mussel, the number of sperm exceeds the number of eggs but in our data the proportion of sperm released relative to eggs is higher at early time points than towards the end of the spawning season. The interaction of environmental and biological factors in synchronising reproduction in both sexes of mussel is likely to be complex and factors introducing asynchrony, may have adverse effects on mussel reproductive productivity. Such factors may be climate change or ocean acidification which has been shown to slow the development of larval forms of mussels [60], [61]. Mussels are of considerable ecological importance and fulfill environmental services so reduced productivity would have adverse environmental effects. Additionally mussels are of economic importance in aquaculture and alterations in the timing of reproduction will have implications for settlement of larvae on culture ropes.

In conclusion this study has provided further evidence for the accuracy and utility of our molecular technique using VERL and VCL for sex determination in Mytilus edulis and we presume this will be applicable in other Mytilus species. This method will be of use in environmental monitoring programmes allowing sex to be determined rapidly so that sex-specific factors in contaminant accumulation or biomarker response do not confound the results. We have defined the reproductive cycle in terms of cyclic variation in transcripts for VERL and VCL and shown that these agree with histological data. Further the observation that these transcripts are present in the gametes provides a rapid method to assess the extent of spawning in any individual/population of mussels. Such information will be of considerable value to mussel growers informing them of suitable times to deploy their ropes for spat settlement.

## Supporting Information

Figure S1 **Variation of Actin between male and female samples over the sampling period.** The graph is plotted with the mean Ct Values (+/− SEM) of actin of male and female samples over the sampling period. It is noticed that the actin for the same amount of cDNA used is not varying between sexes and various gonadal reproductive stages. The Mann-Whitney test for significance had a p value >0.05 for all the male and female samples when compared to female February 2009 samples and thus confirming that there is no significant difference in the variation of actin over the sampling period.
(TIF)

Table S1 **Numbers of male and female animals each month as determined by qPCR (A) and Histology (B).** The number of animals collected and sexed using the qPCR method each month are reported (A). It is seen that the total number of females is higher than males over the sampling period. A total of 312 mussels were collected over 13 months and ∼60% were females and ∼38% were males and ∼2% were undefined. The undefined samples are due to qPCR failures including poor signal for reference gene. Histology results for each month for the matching animals are also shown (B). It is seen that the number of females is higher than males over the sampling period. A total of 312 mussels were collected over 13 months and 40% were females and ∼32% were males and 28% were undefined. It has to be noted that the large number of undefined animals occur during the months that the animals are ‘spent’.
(DOC)

Table S2 **First 5 hits of Blastx search (A) and Relative Expression (B) of H5 cryptic female specific transcript.** The H5 sequence of ≈1100 bp (GeneBank JX297444) was analysed by BLASTx and resulted in the hits shown (A). None of these gave satisfactory results and matches were <20% identical and had high e values in the region of e-07. BLASTn searches found no significant matches. The sequence matches a cryptic, unidentified entry on MytiBase (MGC03205 e = 0.0). The relative expression of H5 in female and male mantle with respect to Feb’09 female H5 samples have been tabulated (B). The relatively low level of expression of H5 in males provides evidence that H5 is a female-specific gene.
(DOC)

Table S3 **Actin Ct values for spawning experiment.** The actin values for male and female gamete and artificially spawned mantle is tabulated. It has to be noted the level of actin is invariant with gametes or mantle and between the two sex as well.
(DOC)

Table S4 **Average Ct values for each month.** The mean Ct values of VCL, VERL and Actin in male and female samples are tabulated in Table S3 and it is seen that the VCL in male is always lower than VERL in male and vice versa, VERL in females are always lower than VCL. It is also noted that the Actin levels are constant throughout the experimental periods in both male and female samples.
(DOC)
