# Supplementary figures and images for: Annual Variation in the Levels of Transcripts of Sex-Specific Genes in the Mantle of the Common Mussel, Mytilus edulis
Source: PLoS One. 2012 Nov 30;7(11):e50861. doi: 10.1371/journal.pone.0050861 (PMC3511322; doi:10.1371/journal.pone.0050861)

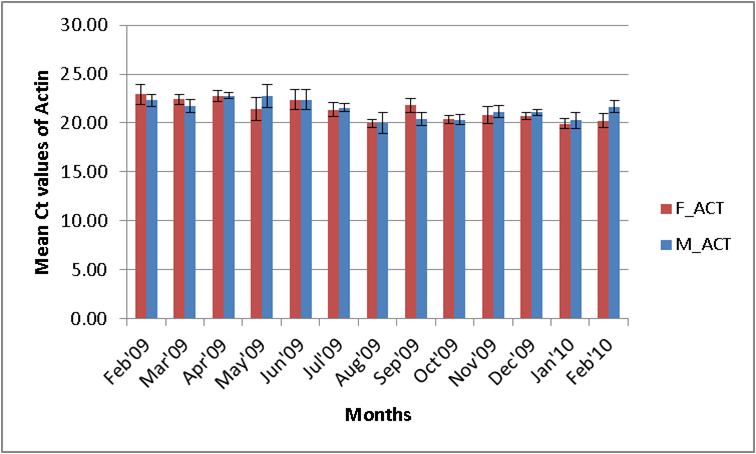

Supplement: Figure S1 — Variation of Actin between male and female samples over the sampling period. The graph is plotted with the mean Ct Values (+/− SEM) of actin of male and female samples over the sampling period. It is noticed that the actin for the same amount of cDNA used is not varying between sexes and various gonadal reproductive stages. The Mann-Whitney test for significance had a p value >0.05 for all the male and female samples when compared to female February 2009 samples and thus confirming that there is no significant difference in the variation of actin over the sampling period. (TIF) [file pone.0050861.s001.tif]
