# Supplementary material for: Annual Variation in the Levels of Transcripts of Sex-Specific Genes in the Mantle of the Common Mussel, Mytilus edulis
Source: PLoS One. 2012 Nov 30;7(11):e50861. doi: 10.1371/journal.pone.0050861 (PMC3511322; doi:10.1371/journal.pone.0050861)
Supplement: Table S1 — Numbers of male and female animals each month as determined by qPCR (A) and Histology (B). The number of animals collected and sexed using the qPCR method each month are reported (A). It is seen that the total number of females is higher than males over the sampling period. A total of 312 mussels were collected over 13 months and ∼60% were females and ∼38% were males and ∼2% were undefined. The undefined samples are due to qPCR failures including poor signal for reference gene. Histology results for each month for the matching animals are also shown (B). It is seen that the number of females is higher than males over the sampling period. A total of 312 mussels were collected over 13 months and 40% were females and ∼32% were males and 28% were undefined. It has to be noted that the large number of undefined animals occur during the months that the animals are ‘spent’. (DOC) [file pone.0050861.s002.doc]

**Table S1: Numbers of male and female animals each month as determined by qPCR (A) and Histology (B).**

**A**

| **Month** | **Female** | **Male** | **Undefined** | **Total** |
| --- | --- | --- | --- | --- |
| **February'09** | 9 | 14 | 1 | 24 |
| **March'09** | 11 | 10 | 3 | 24 |
| **April'09** | 10 | 14 | 0 | 24 |
| **May'09** | 17 | 7 | 0 | 24 |
| **June'09** | 18 | 6 | 0 | 24 |
| **July'09** | 20 | 2 | 2 | 24 |
| **August'09** | 19 | 5 | 0 | 24 |
| **September'09** | 16 | 8 | 0 | 24 |
| **October'09** | 7 | 17 | 0 | 24 |
| **November'09** | 16 | 8 | 0 | 24 |
| **December'09** | 12 | 11 | 1 | 24 |
| **January'10** | 14 | 10 | 0 | 24 |
| **February'10** | 17 | 6 | 1 | 24 |
| **Grand Total** | 186 | 118 | 8 | **312** |

**B**

| **Month** | **Female** | **Male** | **Undefined** | **Total** |
| --- | --- | --- | --- | --- |
| **February'09** | 10 | 14 | 0 | 24 |
| **March'09** | 11 | 13 | 0 | 24 |
| **April'09** | 10 | 14 | 0 | 24 |
| **May'09** | 10 | 6 | 8 | 24 |
| **June'09** | 4 | 5 | 15 | 24 |
| **July'09** | 3 | 1 | 20 | 24 |
| **August'09** | 3 | 2 | 19 | 24 |
| **September'09** | 7 | 3 | 14 | 24 |
| **October'09** | 10 | 6 | 8 | 24 |
| **November'09** | 14 | 8 | 2 | 24 |
| **December'09** | 12 | 12 | 0 | 24 |
| **January'10** | 14 | 10 | 0 | 24 |
| **February'10** | 17 | 7 | 0 | 24 |
| **Grand Total** | 125 | 101 | 86 | **312** |

The number of animals collected and sexed using the qPCR method each month are reported (A). It is seen that the total number of females is higher than males over the sampling period. A total of 312 mussels were collected over 13 months and ~60% were females and ~38% were males and ~2% were undefined. The undefined samples are due to qPCR failures including poor signal for reference gene. Histology results for each month for the matching animals are also shown (B). It is seen that the number of females is higher than males over the sampling period. A total of 312 mussels were collected over 13 months and 40% were females and ~32% were males and 28% were undefined. It has to be noted that the large number of undefined animals occur during the months that the animals are ‘spent’.
