# Supplementary material for: Annual Variation in the Levels of Transcripts of Sex-Specific Genes in the Mantle of the Common Mussel, Mytilus edulis
Source: PLoS One. 2012 Nov 30;7(11):e50861. doi: 10.1371/journal.pone.0050861 (PMC3511322; doi:10.1371/journal.pone.0050861)
Supplement: Table S2 — First 5 hits of Blastx search (A) and Relative Expression (B) of H5 cryptic female specific transcript. The H5 sequence of ≈1100 bp (GeneBank JX297444) was analysed by BLASTx and resulted in the hits shown (A). None of these gave satisfactory results and matches were <20% identical and had high e values in the region of e-07. BLASTn searches found no significant matches. The sequence matches a cryptic, unidentified entry on MytiBase (MGC03205 e = 0.0). The relative expression of H5 in female and male mantle with respect to Feb’09 female H5 samples have been tabulated (B). The relatively low level of expression of H5 in males provides evidence that H5 is a female-specific gene. (DOC) [file pone.0050861.s003.doc]

**Table S2: First 5 hits of Blastx search (A) and Relative Expression (B) of H5 cryptic female specific transcript.**

| Accession | Description | Max Score | Query Coverage | E Value | Max - iden |
| --- | --- | --- | --- | --- | --- |
| [XP_003443185.1](http://www.ncbi.nlm.nih.gov/protein/348511305?report=genbank&log$=prottop&blast_rank=1&RID=ZW24R1YE01S) | PREDICTED: hypothetical protein LOC100701357 [Oreochromis niloticus] | 60.5 | 61% | 3e-07 | 20% |
| [NP_001017910.1](http://www.ncbi.nlm.nih.gov/protein/62955791?report=genbank&log$=prottop&blast_rank=2&RID=ZW24R1YE01S) | rapunzel 4 [Danio rerio] | 60.1 | 61% | 4e-07 | 21% |
| [XP_003443186.1](http://www.ncbi.nlm.nih.gov/protein/348511307?report=genbank&log$=prottop&blast_rank=3&RID=ZW24R1YE01S) | PREDICTED: hypothetical protein LOC100701629 [Oreochromis niloticus] | 58.9 | 67% | 9e-07 | 20% |
| [NP_001139570.1](http://www.ncbi.nlm.nih.gov/protein/225735555?report=genbank&log$=prottop&blast_rank=4&RID=ZW24R1YE01S) | uncharacterized protein LOC100000883 [Danio rerio] | 56.6 | 58% | 5e-06 | 19% |
| XP_003443187.1 | PREDICTED: hypothetical protein LOC100701903 [Oreochromis niloticus] | 56.2 | 69% | 6e-06 | 18% |

**A**

**B**

| RE | **Female** | **Male** |
| --- | --- | --- |
| **Feb'09** | 1.00 | 0.00199 |
| **Mar'09** | 0.63 | 0.00467 |
| **Apr'09** | 0.60 | 0.00132 |
| **May'09** | 0.41 | 0.01288 |
| **June'09** | 0.22 | 0.00115 |
| **Jul'09** | 0.01 | 0.00029 |
| **Aug'09** | 0.00 | 0.00162 |
| **Sep'09** | 0.09 | 0.00433 |
| **Oct'09** | 0.09 | 0.00035 |
| **Nov'09** | 0.14 | 0.00061 |
| **Dec'09** | 0.24 | 0.00006 |
| **Jan'10** | 0.45 | 0.00069 |
| **Feb'10** | 0.91 | 0.00124 |

The H5 sequence of ≈1100bp (GeneBank JX297444) was analysed by BLASTx and resulted in the hits shown (A). None of these gave satisfactory results and matches were <20% identical and had high e values in the region of e-07. BLASTn searches found no significant matches. The sequence matches a cryptic, unidentified entry on MytiBase (MGC03205 e = 0.0). The relative expression of H5 in female and male mantle with respect to Feb’09 female H5 samples have been tabulated (B). The relatively low level of expression of H5 in males provides evidence that H5 is a female-specific gene.
