# Supplementary material for: Annual Variation in the Levels of Transcripts of Sex-Specific Genes in the Mantle of the Common Mussel, Mytilus edulis
Source: PLoS One. 2012 Nov 30;7(11):e50861. doi: 10.1371/journal.pone.0050861 (PMC3511322; doi:10.1371/journal.pone.0050861)
Supplement: Table S3 — Actin Ct values for spawning experiment. The actin values for male and female gamete and artificially spawned mantle is tabulated. It has to be noted the level of actin is invariant with gametes or mantle and between the two sex as well. (DOC) [file pone.0050861.s004.doc]

Table S3: Actin Ct values for spawning experiment

| Actin Ct | Male | Female |
| --- | --- | --- |
| Gamete | 18.923 | 19.241 |
| Mantle | 19.012 | 18.792 |

The actin values for male and female gamete and artificially spawned mantle is tabulated. It has to be noted the level of actin is invariant with gametes or mantle and between the two sex as well.
