# Supplementary material for: Annual Variation in the Levels of Transcripts of Sex-Specific Genes in the Mantle of the Common Mussel, Mytilus edulis
Source: PLoS One. 2012 Nov 30;7(11):e50861. doi: 10.1371/journal.pone.0050861 (PMC3511322; doi:10.1371/journal.pone.0050861)
Supplement: Table S4 — Average Ct values for each month. The mean Ct values of VCL, VERL and Actin in male and female samples are tabulated in Table S3 and it is seen that the VCL in male is always lower than VERL in male and vice versa, VERL in females are always lower than VCL. It is also noted that the Actin levels are constant throughout the experimental periods in both male and female samples. (DOC) [file pone.0050861.s005.doc]

**Table S4: Average Ct values for each month**

|  | **Female** |  |  | **Male** |  |  |
| --- | --- | --- | --- | --- | --- | --- |
| **Ct Values** | **F_VCL** | **F_VERL** | **F_ACT** | **M_VCL** | **M_VERL** | **M_ACT** |
| **Feb'09** | 28.04 | 19.38 | 22.92 | 19.57 | 26.98 | 22.30 |
| **Mar'09** | 31.67 | 18.03 | 22.40 | 17.82 | 27.14 | 21.72 |
| **Apr'09** | 28.78 | 18.02 | 22.80 | 19.39 | 27.10 | 22.77 |
| **May'09** | 32.05 | 19.94 | 21.40 | 20.55 | 27.24 | 22.75 |
| **Jun'09** | 30.22 | 21.35 | 22.35 | 22.83 | 28.37 | 22.36 |
| **Jul'09** | 30.35 | 24.54 | 21.35 | 24.75 | 27.56 | 21.57 |
| **Aug'09** | 28.93 | 21.60 | 19.97 | 26.12 | 29.76 | 20.00 |
| **Sep'09** | 30.17 | 20.51 | 21.81 | 21.47 | 28.45 | 20.36 |
| **Oct'09** | 28.32 | 17.92 | 20.36 | 19.77 | 27.65 | 20.35 |
| **Nov'09** | 26.50 | 18.29 | 20.80 | 19.82 | 27.45 | 21.16 |
| **Dec'09** | 26.89 | 17.51 | 20.72 | 18.99 | 27.60 | 21.10 |
| **Jan'10** | 27.27 | 14.91 | 19.91 | 18.36 | 26.20 | 20.29 |
| **Feb'10** | 29.17 | 15.47 | 20.24 | 20.21 | 26.69 | 21.65 |

The mean Ct values of VCL, VERL and Actin in male and female samples are tabulated in Table S3 and it is seen that the VCL in male is always lower than VERL in male and vice versa, VERL in females are always lower than VCL. It is also noted that the Actin levels are constant throughout the experimental periods in both male and female samples.
